# Report of Recombinant Norovirus GII.g/GII.12 in Beijing, China

**DOI:** 10.1371/journal.pone.0088210

**Published:** 2014-02-05

**Authors:** Shaowei Sang, Zhongtang Zhao, Jijiang Suo, Yubin Xing, Ning Jia, Yan Gao, Lijun Xie, Mingmei Du, Bowei Liu, Shiwang Ren, Yunxi Liu

**Affiliations:** 1 Department of Epidemiology and Health Statistics, School of Public Health, Shandong University, Ji'nan, Shandong, China,; 2 Department of Infection Management and Disease Control, Chinese People's Liberation Army (PLA) General Hospital, Beijing, China; University of Oxford, Viet Nam

## Abstract

**Background:**

Norovirus (NoV) has been recognized as the most important cause of nonbacterial acute gastroenteritis affecting all age group people in the world. Genetic recombination is a common occurance in RNA viruses and many recombinant NoV strains have been described since it was first reported in 1997. However, the knowledge of recombinant NoV in China is extremely limited.

**Methods:**

A total of 685 stool specimens were tested for NoV infection from the acute gastroenteritis patients who visited one general hospital in Beijing from April 2009 to November 2011. The virus recombination was identified by constructing phylogenetic trees of two genes, further SimPlot and the maximum chi-square analysis.

**Results:**

The overall positive rate was 9.6% (66/685). GII.4 New Orleans 2009 and GII.4 2006b variants were the dominant genotype. Four GII.g/GII.12 and one GII.12/GII.3 recombinant strains were confirmed, and all derived from adult outpatients. The predictive recombination point occurred at the open reading frame (ORF)1/ORF2 overlap.

**Conclusions:**

The GII.g ORF1/GII.12ORF2 recombinant has been reported in several countries and it was the first report of this recombinant in China.

## Introduction

*Norovirus* (NoV), of the genus Norovirus, family *Caliciviridae*, was first detected in samples derived from an outbreak at a school in Norwalk, OH, in 1968 and the virus was observed under the immune electron microscopy (IEM) in 1972[1]. NoV has been recognized as the most important cause of nonbacterial acute gastroenteritis in both developed and developing countries, affecting both children and adults[2]. It is estimated that there are over 23 million NoV gastroenteritis each year[3]. In immunosuppressive conditions or multi-organ dysfunctional patients, infection of NoV can not only prolong the hospitalization but also induces some complications and sometimes even death[4].

NoV is a nonenveloped positive sense, single-stranded RNA virus with a genome of approximate 7.5 kb[5]. The genome has three open reading frames (ORFs) with ORF1 and ORF2 overlapping about twenty base pair nucleotides[6]. ORF1 encodes non-structural protein including RNA-dependent RNA polymerase (RdRp). ORF2 encodes major capsid protein (VP1) that contains a N-terminal arm, a shell or S-domain and a protrusion or P-domain. The P-domain is divided into 2 sub-domains called P1 and P2, the latter corresponding to the most variable region of the capsid. ORF3 encodes minor capsid protein (VP2)[7], [8]. NoV can be grouped into five genogroups, GI–GV and only GI, GIIand GIV can infect humans, of which GIIis the most common. Each genogroup includes several genotypes [9], [10], [11].

Genetic recombination is common in RNA viruses, and several recombinant NoV strains have been described since it was first reported in 1997[12]. In the previous reports, NoV recombinant strains have caused several acute gastroenteritis outbreaks[13]. As recombination may allow the virus to increase its fitness or escape herd immunity, it is important to perform surveillance for recombination in norovirus. The recombination of NoV occurs usually at the overlapping area of ORF1 and ORF2, thus, a recombinant NoV can clustered within 2 distinct groups when the capsid and polymerase regions of the genome are subjected to phylogenetic analysis[6].It would be questionable to classify genotype only on the basis of capsid sequence or polymerase sequence. The first recombinant NoV isolate detected in China was 146/Kunming/04/China (GenBank accession number DQ304651) in 2006[14]. From then on, some studies began to use genetic sequencing plus Clustal and Simplot analysis to identify recombinant virus in China. Some new recombinant NoV strains, such as GII.16/GII.2 [15], GII.b/GII.18 [16] were reported, however, the knowledge on recombinant NoV is extremely limited in China. In this study, we aimed to identify the recombinant NoV both from adult and child patients who visited Chinese PLA General Hospital in Beijing.

## Materials and Methods

### Ethics Statement

The study protocol and consent procedure were approved by the Ethical and Scientific Review Subcommittee of the Ministry of Public Health of China. Informed oral consent was obtained from the parents or child guardians before samples were collected. Oral consent was deemed more suitable for this study due to the high rate of illiteracy in the study population. After the consent was given, personal details, as well as, epidemiological data were recorded in a paper file.

### Specimens

Stool samples were collected under one gastroenteritis surveillance program at Chinese PLA General Hospital in Beijing from April 2009 to November 2011. Both outpatients and inpatients were enrolled, who presented with three or more liquid stools in 24 hours or vomiting, or two or more of the following signs: fever, abdominal pain, malaise and nausea. A total of 685 patients were included. The median age was 34 years old (range 1–91) and 365 (53%) were male. Stool specimens were obtained within 24 hrs hospital admission and stored at −70°C until use.

### RNA Extraction

Viral RNA was extracted from 10% stool suspensions in phosphate-buffered saline (pH 7.2) using Viral Nucleic Acid Extraction KitIIas the manufacturer's instructions, and was stored at −70°C.

### RT-PCR and Sequence Analysis

To amplify NoV GI partial capsid gene (330 bp), the primer set G1SKF/G1SKR was used[17]. To amplify the same region of NoV GII (387 bp), the primer set G2SKF/G2SKR was first used [17], then the primer set CoG2F/G2SKR[18] was used for RT-PCR negative specimen to increase the detection sensitivity. To amplify the fragment of the RdRp gene (326 bp), the primer set JV12/JV13[19] was used. The ORF1/ORF2 overlap region (1055 bp) was amplified when one sample was classified into 2 distinct groups using partial capsid and RdRp gene phylogenetic analysis respectively. To produce a product of this region, the primer set JV12/G2SKR was used. For all the RT-PCR assays, RNA extraction, the reagent setup, amplification, and agarose gel electrophoresis were performed in separate rooms, and negative and no template controls (distilled water) were concurrently included in each amplification. RT-PCR products were sequenced directly.

### Recombination Analysis

To identify recombinant, we performed phylogenetic analysis plus SimPlot3 and maximum chi-square analysis. MEGA Version 5.1 software was used for sequence alignment and phylogenetic analysis. Sites with ambiguous alignments were removed before phylogenetic analysis. The phylogenetic tree was calculated by Maximum Likelihood method (ML). The Substitution Model was General Time Reversible model (GTR) and ML Heuristic Method was Nearest-Neighbor-Interchange (NNI). The stability of the tree was evaluated by bootstrap analysis with 1000 replications. SimPlot3 analysis was used to confirm putative recombinant strains and to identify putative recombination point as previous reports[20], [21]. The maximum chi-square method was the third analysis to confirm recombinants. The strains were defined as recombinants if the crossover event was found to be significant (*p*<0.01)[22].

Nucleotide Sequence Accession Numbers: Some nucleotide sequences reported in this article had been deposited in GenBank (Nucleotide sequence accession numbers JQ889812 to JQ889817 and JQ899442).

## Results

A total of 685 fecal specimens were collected, including 167 specimens from 2009, 160 ones from 2010 and 358 ones from 2011. The overall prevalence of the NoV infection was 9.6% (66/685), and the positive rate was 3.6% (6/167), 8.8% (14/160), and 12.8% (46/358) each year respectively. No significant difference was observed between adult and child patients (9.5% VS 13.4%, *p* = 0.6).

**Figure 1:**
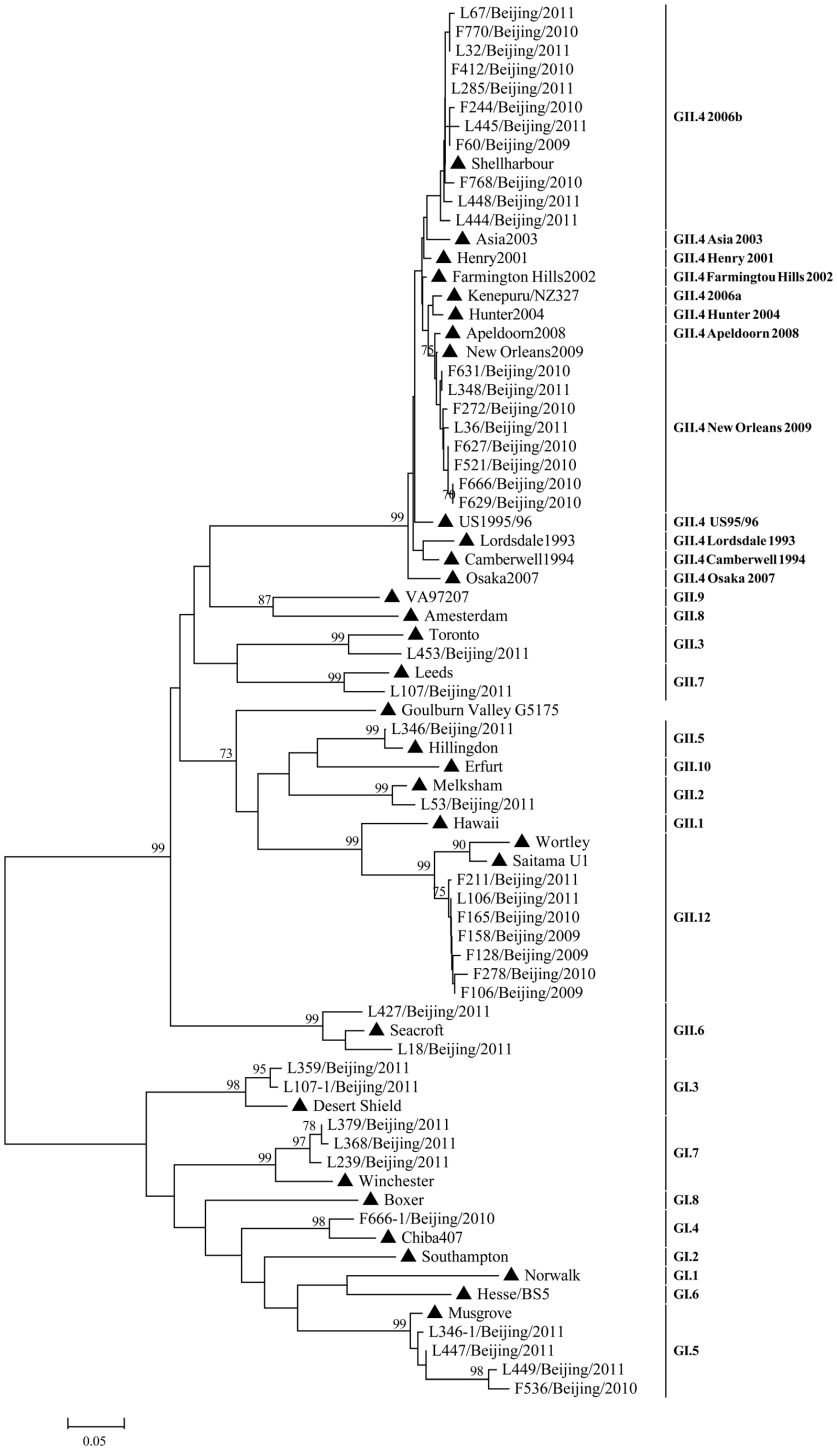
The Maximum Likelihood (ML) phylogenetic tree was constructed with partial nucleotide sequences of ORF2(265 bp). ▴ represented reference strains including Shellharbour (EF684915), Asia 2003 (DQ369797), Henry 2001(EU310927), Farmington Hill 2002 (AY502023), Kenepuru/NZ327 (EF187497), Hunter 2004 (DQ078814), New Orleans 2009 (GQ845367), Apeldoorn2008 (HQ009513), US 1995/96 (AY741811), Osaka 2007 (AB541319), Camberwell 1994 (AF145896), Lordsdale 1993 (X86557), VA97207 (AY038599), Amsterdam (AF195848), Leeds(AJ277608), Toronto (U02030), Hillingdon (AJ277607), Melksham (X81879), Erfurt (AF427118), Hawaii (U07611), Wortley (AJ277618), Saitama U1 (AB039775), Seacroft (AJ277620), DesertShield(U04469), Winchester(AJ277609), Boxer (AF538679), Chiba407(AB042808), Norwalk (M87661), Southampton (L07418), Hesse/BS5 (AF093797), Musgrove(AJ277614), Goulburn Vally G5175 (DQ379714).

**Figure 2:**
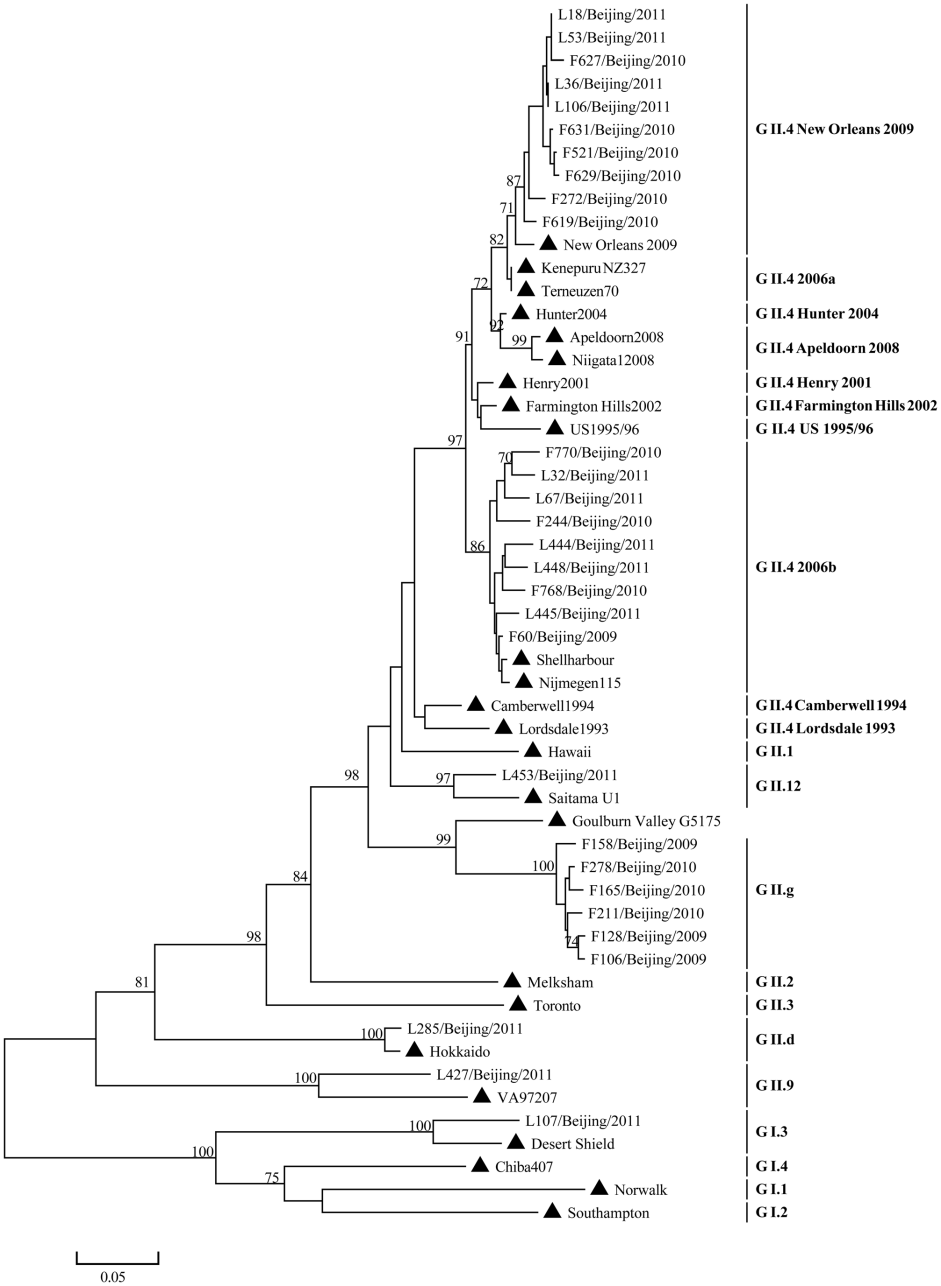
The Maximum Likelihood (ML) phylogenetic tree was constructed with partial nucleotide sequences of ORF1(326 bp) using the primers of JV12/JV13. ▴ represented reference strains including New Orleans 2009 (GQ845367), Kenepuru NZ327 (EF187497), Terneuzen70 (EF126964), Hunter 2004 (DQ078814), Apeldoorn2008 (HQ009513), Niigata12008 (AB541310), Henry 2001(EU310927), US 1995/96 (AY741811), Farmington Hill 2002 (AY502023), Shellharbour (EF684915), Nijimegen115 (EF126966),Camberwell 1994 (AF145896), Lordsdale 1993 (X86557), Hawaii (U07611), Saitama U1 (AB039775), Goulburn Vally G5175 (DQ379714), Melksham (X81879), Toronto (U02030),Hokkaido (AB212306), DesertShield (U04469), Norwalk (M87661), Southampton (L07418), Chiba407 (AB042808),VA97207 (AY038599).

Using the partial gene of VP1(ORF2) for classification, 42 specimens were successfully genotyped, including 10 (23.8%) G I (2 GI.3, 1 GI.4, 4 GI.5, 3 GI.7) and 32(76.2%) GII (1 GII.2, 1 GII.3, 19 GII.4, 1 GII.5, 2 GII.6, 1 GII.7, 7 GII.12) (**Figures 1**). GII.4 New Orleans 2009 and GII.4 2006b variants were the dominant genotypes. While using the partial gene of RdRp (ORF1) to classify above 42 specimens, 29 strains were classified into the same genotype as VP1 (**Figure. 2**), but 12 were not unanimous, indicating pupative recombination or mixed infection (1 sample was failed to be further classified due to limited template).

In order to rule out possible mixed infection and to map the recombination position of above 12 NoV strains, amplicons encompassing the highly recombination-prone ORF1-ORF2 junction region were generated with primers JV12/G2SKR and were further sequenced. The amplicon was 1009 bp and located at the position 4327–5336 corresponding to the reference strain Hawaii (GenBank accession number U07611). Four NoV strains (F106, F158, F128, F278) showed 99% similarity with those sequences of published GII.g/GII.12 recombinant strains (Seoul/0448/2009/KOR (GenBank accession number HM635104), StGeorge/NSW199U/2008/AU (GenBank accession number GQ845370)), and they were clustered with Goulburn Valley G5175B/1983/AUS (GenBank accession number DQ379714) (recombinant of GII.g/GII.13) and Saitama U1(GenBank accession number AB039775)(GII.12) by phylogenetic calculation of RdRp and VP1genes respectively (**Figure 2** **,** **1**). Another strain, L453, was grouped with Saitama U1(GenBank accession number AB039775) (GII.12) and Toronto (GenBank accession number U02030)(GII.3) (**Figure 2** **,** **1**). Two other strains, L18 and L53, were suspected as mixed infections (**Table 1**).

**Table 1:** The inconsistant results of specimens genotyping with the partial gene of RdRp or VP1 of NoV.

Accession Number	Specimens	Separate genotyping results		Analyzing of JV12/G2SKR products
		RdRp	VP1	
JQ889814	F158/Beijng/2009	GII.g	GII.12	recombination
JQ899442	F278/Beijng/2010	GII.g	GII.12	recombination
JQ889815	F128/Beijng/2009	GII.g	GII.12	recombination
JQ889812	F106/Beijng/2009	GII.g	GII.12	recombination
JQ889813	L453/Beijng/2011	GII.12	GII.3	recombination
JQ889816	L18/Beijng/2011	GII.4	GII.6	Mixed infection
JQ889817	L53/Beijng/2011	GII.4	GII.2	Mixed infection
	F165/Beijng/2009	GII.g	GII.12	--
	F211/Beijng/2009	GII.g	GII.12	--
	L427/Beijng/2011	GII.9	GII.6	--
	L285/Beijng/2011	GII.d	GII.4	--
	L106/Beijng/2011	GII.4	GII.12	--

Note: ‘--’means the specimen was not successfully genotyped.

**Figure 3:**
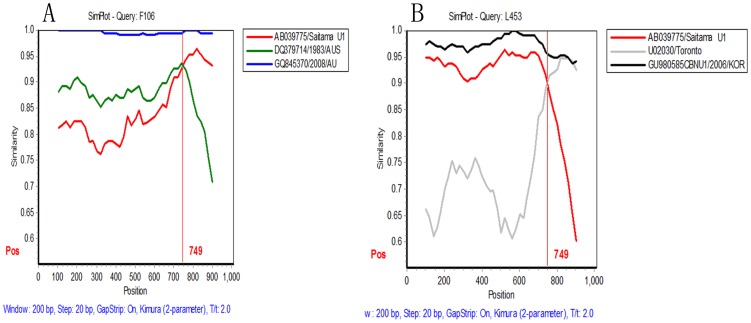
Similarity plot of NoV GII recombinants. The graph represents as a decimal identity of the two putative parental strains with their respective recombinant(The fragment was 1055 bp). The window size is 200(i.e. where the lines cross) is the predicted site of recombination. A.The red line, green line, blue line represent AB039775/Saitama U1, DQ379714/1983/AUS, GQ845370/2008/AU respectively. B. The red line, grey line and dark line represent AB039775/Saitama U1,U02030/Toronto and GU980585/CBNU1/2006/KOR respectively.

Then we chose Goulburn Valley G5175B/1983/AUS (GenBank accession number DQ379714) and Saitama U1(GenBank accession number AB039775) as the parental strains of F106, F158, F128, F278, and chose Saitama U1(GenBank accession number AB039775) and Toronto (GenBank accession number U02030) as the parental strains of L453. SimPlot analysis indicated that F106, F158, F128, F278 were recombinants of GII.g/GII.12 and L453 was recombinant of GII.12/GII.3. The predictive recombination point of these recombinants was 749, which was -9 corresponding to Hawaii(GenBank accession numberU07611)(the ORF1 and ORF2 overlap of U07611 is 5085–5104 and we set 5085 as 1,with numbering increasing towards the 3′ end and decreasing towards the 5′ end of the genome) (**Figure. 3**). It implied the recombination point took place at the ORF1/ORF2 overlap. Using maximum chi-square method, L453 was recombinant of GII.12/GII.3 and the crossover event was significant (*p* = 3.902×10^−9^), but the break point was 779 which was different from the SimPlot analysis (749).

Four GII.g/GII.12 recombinant strains were all detected from adult outpatients, including 2 females and 2 males. One GII.12/GII.3 recombinant strain was identified from one female outpatient aged 47 years old.

## Discussion

With the development of molecular biology techniques, NoV was considered as the leading cause of food-borne disease and acute non-bacterial gastroenteritis worldwide[23]. Because NoV can be genotyped with either RdRp or VP1 gene, the results are sometimes different from the two methods [22], [24], [25], which is best explained by recombination or mixed infection. As most of the recombination point located at the ORF1/ORF2 overlap, most researches target partial sequences of RdRp and VP1 gene including the overlapping area to identify the genotype. In this study, we reported four GII.g/GII.12 recombinant strains and one GII.12/GII. 3 recombinant strain using three analysis methods.

The parental strains of GII.g/GII.12 were Goulburn Valley G5175B/1983/AUS (GenBank accession number DQ379714) and Saitama U1(GenBank accession number AB039775). Goulburn Valley G5175B/1983/AUS (GenBank accession number DQ379714) was the recombinant of GII.g ORF1 and GII.13ORF2, and Saitama U1(GenBank accession number AB039775) was genotyped as GII.12 which was the recombinant of GII.4 ORF1 and GII.12 ORF2 in some publications [26], [27]. The recombination of GII.g/GII.12 was first detected in sporadic cases in Australia in 2008 and the recombinant caused outbreak in New Zealand in the same year[28]. The recombinant also caused outbreak in America[29], Belgium[30], Korea[31] and Italy[26]. However it is the first report of GII.g/GII.12 recombinant in China. Giovanni et al [26] estimated evolution rate of partial ORF2 sequences(region C) of GII.g/GII.12 was 3.7×10^−3^ (SD 7.2×10^−5^) nt substitutions/site/year using Bayesian phylogenetic reconstructions. This evolution rate was almost consistent with GII.4 (4.3−9.0×10^−3^)[32], which may explain the reason for the broad distribution of this recombinant strain. GII.12/GII.3 recombinant has been already prevalent in China. China CDC announced that the prevalence of GII.12/GII.3 was second to GII.4 2006b in 2006–2007 and the recombinant was detected in several provinces, such as Hebei, Henan, Jilin, Shanxi, Shannxi, Shanghai[33].

NoV recombination can greatly affect genotyping, confuse molecular epidemiologic studies, and have major implications in viral vaccine design. A continuous monitoring of the virus genetic evolution is warranted. With more and more new recombinant NoV strains being identified, we highlight the importance on surveillance of NoV infection in the hospital using two amplification protocols (ORF1 and ORF2) to identify recombination events among NoV genotypes or genogroups.

## References

[1] Kapikian AZ, Wyatt RG, Dolin R, Thornhill TS, Kalica AR, et al (1972) Visualization by immune electron microscopy of a 27-nm particle associated with acute infectious nonbacterial gastroenteritis. J Virol 10: 1075–1081. 4117963 10.1128/jvi.10.5.1075-1081.1972 PMC356579

[2] Yang Y, Xia M, Tan M, Huang P, Zhong W, et al (2010) Genetic and phenotypic characterization of GII-4 noroviruses that circulated during 1987 to 2008. J Virol 84: 9595–9607. 20592096 10.1128/JVI.02614-09 PMC2937647

[3] Mead PS, Slutsker L, Dietz V, McCaig LF, Bresee JS, et al (1999) Food-related illness and death in the United States. Emerg Infect Dis 5: 607–625. 10511517 10.3201/eid0505.990502 PMC2627714

[4] Harris JP, Edmunds WJ, Pebody R, Brown DW, Lopman BA (2008) Deaths from norovirus among the elderly, England and Wales. Emerg Infect Dis 14: 1546–1552. 18826817 10.3201/eid1410.080188 PMC2609872

[5] Xi JN, Graham DY, Wang KN, Estes MK (1990) Norwalk virus genome cloning and characterization. Science 250: 1580–1583. 2177224 10.1126/science.2177224

[6] Bull RA, Hansman GS, Clancy LE, Tanaka MM, Rawlinson WD, et al (2005) Norovirus recombination in ORF1/ORF2 overlap. Emerg Infect Dis 11: 1079–1085. 16022784 10.3201/eid1107.041273 PMC3371806

[7] Bertolotti-Ciarlet A, Crawford SE, Hutson AM, Estes MK (2003) The 3' end of Norwalk virus mRNA contains determinants that regulate the expression and stability of the viral capsid protein VP1: a novel function for the VP2 protein. J Virol 77: 11603–11615. 14557646 10.1128/JVI.77.21.11603-11615.2003 PMC229252

[8] Green J, Vinje J, Gallimore CI, Koopmans M, Hale A, et al (2000) Capsid protein diversity among Norwalk-like viruses. Virus Genes 20: 227–236. 10949950 10.1023/a:1008140611929

[9] Ando T, Noel JS, Fankhauser RL (2000) Genetic classification of "Norwalk-like viruses. J Infect Dis 181 Suppl 2: S336–348. 10804147 10.1086/315589

[10] Zheng DP, Ando T, Fankhauser RL, Beard RS, Glass RI, et al (2006) Norovirus classification and proposed strain nomenclature. Virology 346: 312–323. 16343580 10.1016/j.virol.2005.11.015

[11] Kageyama T, Shinohara M, Uchida K, Fukushi S, Hoshino FB, et al (2004) Coexistence of multiple genotypes, including newly identified genotypes, in outbreaks of gastroenteritis due to Norovirus in Japan. J Clin Microbiol 42: 2988–2995. 15243049 10.1128/JCM.42.7.2988-2995.2004 PMC446284

[12] Hardy ME, Kramer SF, Treanor JJ, Estes MK (1997) Human calicivirus genogroup II capsid sequence diversity revealed by analyses of the prototype Snow Mountain agent. Arch Virol 142: 1469–1479. 9267456 10.1007/s007050050173

[13] Chung JY, Han TH, Park SH, Kim SW, Hwang ES (2010) Detection of GII-4/2006b variant and recombinant noroviruses in children with acute gastroenteritis, South Korea. J Med Virol 82: 146–152. 19950237 10.1002/jmv.21650

[14] Phan TG, Yan H, Li Y, Okitsu S, Muller WE, et al (2006) Novel recombinant norovirus in China. Emerg Infect Dis 12: 857–858. 16710954 10.3201/eid1205.051566 PMC3374456

[15] Wang YH, Zhou DJ, Zhou X, Yang T, Ghosh S, et al (2012) Molecular epidemiology of noroviruses in children and adults with acute gastroenteritis in Wuhan, China, 2007–2010. Arch Virol 157: 2417–2424. 22886184 10.1007/s00705-012-1437-1

[16] Dai YC, Hu GF, Zhang XF, Song CL, Xiang WL, et al (2011) Molecular epidemiology of norovirus gastroenteritis in children in Jiangmen, China, 2005–2007. Arch Virol 156: 1641–1646. 21562879 10.1007/s00705-011-1010-3

[17] Kojima S, Kageyama T, Fukushi S, Hoshino FB, Shinohara M, et al (2002) Genogroup-specific PCR primers for detection of Norwalk-like viruses. J Virol Methods 100: 107–114. 11742657 10.1016/s0166-0934(01)00404-9

[18] Yan H, Yagyu F, Okitsu S, Nishio O, Ushijima H (2003) Detection of norovirus (GI, GII), Sapovirus and astrovirus in fecal samples using reverse transcription single-round multiplex PCR. J Virol Methods 114: 37–44. 14599677 10.1016/j.jviromet.2003.08.009

[19] Vinje J, Koopmans MP (1996) Molecular detection and epidemiology of small round-structured viruses in outbreaks of gastroenteritis in the Netherlands. J Infect Dis 174: 610–615. 8769621 10.1093/infdis/174.3.610

[20] Lole KS, Bollinger RC, Paranjape RS, Gadkari D, Kulkarni SS, et al (1999) Full-length human immunodeficiency virus type 1 genomes from subtype C-infected seroconverters in India, with evidence of intersubtype recombination. J Virol 73: 152–160. 9847317 10.1128/jvi.73.1.152-160.1999 PMC103818

[21] Smith JM (1992) Analyzing the mosaic structure of genes. J Mol Evol 34: 126–129. 1556748 10.1007/BF00182389

[22] Bull RA, Tanaka MM, White PA (2007) Norovirus recombination. J Gen Virol 88: 3347–3359. 18024905 10.1099/vir.0.83321-0

[23] Atmar RL, Estes MK (2006) The epidemiologic and clinical importance of norovirus infection. Gastroenterol Clin North Am 35 : 275––290, viii. 10.1016/j.gtc.2006.03.001 16880066

[24] Ambert-Balay K, Bon F, Le Guyader F, Pothier P, Kohli E (2005) Characterization of new recombinant noroviruses. J Clin Microbiol 43: 5179–5186. 16207981 10.1128/JCM.43.10.5179-5186.2005 PMC1248523

[25] Nakamura K, Iwai M, Zhang J, Obara M, Horimoto E, et al (2009) Detection of a novel recombinant norovirus from sewage water in toyama prefecture, Japan. Jpn J Infect Dis 62: 394–398. 19762994

[26] Giammanco GM, Rotolo V, Medici MC, Tummolo F, Bonura F, et al (2012) Recombinant norovirus GII.g/GII.12 gastroenteritis in children. Infect Genet Evol 12: 169–174. 22067516 10.1016/j.meegid.2011.10.021

[27] Katayama K, Shirato-Horikoshi H, Kojima S, Kageyama T, Oka T, et al (2002) Phylogenetic analysis of the complete genome of 18 Norwalk-like viruses. Virology 299: 225–239. 12202225 10.1006/viro.2002.1568

[28] Eden JS, Bull RA, Tu E, McIver CJ, Lyon MJ, et al (2010) Norovirus GII.4 variant 2006b caused epidemics of acute gastroenteritis in Australia during 2007 and 2008. J Clin Virol 49: 265–271. 20888289 10.1016/j.jcv.2010.09.001

[29] Takanashi S, Wang Q, Chen N, Shen Q, Jung K, et al (2011) Characterization of emerging GII.g/GII.12 noroviruses from a gastroenteritis outbreak in the United States in 2010. J Clin Microbiol 49: 3234–3244. 21752978 10.1128/JCM.00305-11 PMC3165588

[30] Mathijs E, Denayer S, Palmeira L, Botteldoorn N, Scipioni A, et al (2011) Novel norovirus recombinants and of GII.4 sub-lineages associated with outbreaks between 2006 and 2010 in Belgium. Virol J 8: 310. 21682917 10.1186/1743-422X-8-310 PMC3135559

[31] Han TH, Kim CH, Chung JY, Park SH, Hwang ES (2011) Emergence of norovirus GII-4/2008 variant and recombinant strains in Seoul, Korea. Arch Virol 156: 323–329. 21053029 10.1007/s00705-010-0844-4

[32] Siebenga JJ, Lemey P, Kosakovsky Pond SL, Rambaut A, Vennema H, et al (2010) Phylodynamic reconstruction reveals norovirus GII.4 epidemic expansions and their molecular determinants. PLoS Pathog 6: e1000884. 20463813 10.1371/journal.ppat.1000884 PMC2865530

[33] Jin M, Xie HP, Duan ZJ, Liu N, Zhang Q, et al (2008) Emergence of the GII4/2006b variant and recombinant noroviruses in China. J Med Virol 80: 1997–2004. 18814250 10.1002/jmv.21308

